# A Cistanches Herba Fraction/***β***-Sitosterol Causes a Redox-Sensitive Induction of Mitochondrial Uncoupling and Activation of Adenosine Monophosphate-Dependent Protein Kinase/Peroxisome Proliferator-Activated Receptor ***γ*** Coactivator-1 in C2C12 Myotubes: A Possible Mechanism Underlying the Weight Reduction Effect

**DOI:** 10.1155/2015/142059

**Published:** 2015-02-03

**Authors:** Hoi Shan Wong, Jihang Chen, Pou Kuan Leong, Hoi Yan Leung, Wing Man Chan, Kam Ming Ko

**Affiliations:** Division of Life Science, Hong Kong University of Science and Technology, Kowloon, Hong Kong

## Abstract

Previous studies have demonstrated that HCF1, a semipurified fraction of Cistanches Herba, causes weight reduction in normal diet- and high fat diet-fed mice. The weight reduction was associated with the induction of mitochondrial uncoupling and changes in metabolic enzyme activities in mouse skeletal muscle. To further investigate the biochemical mechanism underlying the HCF1-induced weight reduction, the effect of HCF1 and its active component, *β*-sitosterol (BSS), on C2C12 myotubes was examined. Incubation with HCF1/BSS caused a transient increase in mitochondrial membrane potential (MMP), possibly by fluidizing the mitochondrial inner membrane. The increase in MMP was paralleled to an increase in mitochondrial reactive oxygen species (ROS) production. Mitochondrial ROS, in turn, triggered a redox-sensitive induction of mitochondrial uncoupling by uncoupling protein 3 (UCP3). Biochemical analysis indicated that HCF1 was capable of activating an adenosine monophosphate-dependent protein kinase/peroxisome proliferator-activated receptor *γ* coactivator-1 pathway and thereby increased the expression of cytochrome c oxidase and UCP3. Animal studies using mitochondrial recoupler also confirmed the role of mitochondrial uncoupling in the HCF1-induced weight reduction. In conclusion, a HCF1/BSS causes the redox-sensitive induction of mitochondrial uncoupling and activation of AMPK/PGC-1 in C2C12 myotubes, with resultant reductions in body weight and adiposity by increased energy consumption.

## 1. Introduction

Obesity, which is defined as an abnormal accumulation of body fat, has emerged as a public health threat. Regardless of metabolically healthy or metabolically impaired conditions, obesity has been found to be associated with an increased risk of comorbidities and mortality [1]. Central obesity, abnormalities in cholesterol profiles, and elevations in plasma triglyceride levels, all of which are common features of obese individuals, contribute to the high incidence of type 2 diabetes, cardiovascular diseases, noncardiovascular death, and obesity-related cancers in obese individuals [2]. Therefore, reduction in body weight and adiposity has been considered as key for the prevention of obesity-related health consequences.

The management of obesity mainly emphasizes the establishment of an optimal energy balance by increasing energy expenditure and/or reducing energy intake. Lifestyle modifications, including an increase in physical activity and the development of healthy eating patterns, are generally considered as safe approaches to induce weight loss. However, such approaches have generally been found to be inefficient to increase energy expenditure or reduce energy intake [3]. Therefore, strategies based on the use of pharmacological agents are currently under active investigation. In this connection, our previous finding has demonstrated that HCF1, a semipurified fraction of Cistanches Herba (a dried whole plant of* Cistanche deserticola* Y.C. Ma characterized as a “Yang-invigorating” tonic herb in traditional Chinese medicine), was shown to be effective in preventing diet-induced obesity and its associated metabolic abnormalities [4]. The oral administration of HCF1 produced weight reduction in normal diet- (ND-) and high fat diet- (HFD-) fed mice, possibly by the induction of mitochondrial uncoupling in skeletal muscle, with a resultant increase in energy expenditure [4]. However, the mechanism underlying such HCF1-induced mitochondrial uncoupling (and hence the weight reduction) remains unclear.

Mitochondrial uncoupling dissipates proton gradients by introducing an alternative proton conductance pathway across the inner mitochondrial membrane (IMM), with a resultant decrease in mitochondrial membrane potential and mitochondrial ATP generation. This futile cycle of proton transport consumes a high proportion of metabolic energy in various tissues and is responsible for a significant portion of daily energy expenditure, with a subsequent increase in the use of fuel molecules (such as fatty acids), thereby causing weight loss and reduction in adiposity [5, 6]. To confirm the mitochondrial uncoupling effect produced by HCF1, the effects of HCF1 on MMP and UCP3 expression were examined in C2C12 myotubes, which are differentiated muscle cells derived from the thigh muscle of C3H mice. Our aim was to investigate the biochemical mechanism underlying the weight reduction afforded by HCF1. *β*-sitosterol (BSS), an active component of HCF1, was also studied for comparison. The possible involvement of mitochondrial uncoupling in HCF1-induced weight reduction in HFD-fed obese mice was further investigated by the coadministration of HCF1 with a chemical recoupler, 6-ketocholestanol (kCh).

Adenosine monophosphate-dependent protein kinase (AMPK), a ubiquitously expressed serine/threonine protein kinase, constitutes an important hub for the control of cellular energy metabolism. Upon activation, AMPK integrates intra-/extracellular signals and elicits cellular responses through the intermediacy of various cellular signaling cascades [7, 8]. AMPK is capable of modulating cellular metabolism by regulating metabolic enzyme activities via direct phosphorylation. AMPK also produces long-term effects at a transcriptional level via its phosphorylation of peroxisome proliferator-activated receptor *γ* coactivator-1 (PGC-1), which leads to adaptive changes in gene expression related to energy metabolism and mitochondrial function [9, 10]. In this regard, we also examined the effect of HCF1/BSS on the AMPK/PGC-1 signaling pathway in C2C12 myotubes.

## 2. Materials and Methods

### 2.1. Herbal Extraction

Cistanches Herba, the dried whole plant of* Cistanche deserticola* Y.C. Ma (Orobanchaceae), was purchased from a local herbal dealer (Lee Hoong Kee). The herb was authenticated by the supplier and a voucher specimen (HKUST00301) was deposited in the Division of Life Science, the Hong Kong University of Science and Technology (HKUST). A Cistanches Herba extract was obtained by ethanol extraction of ground herbal material by heating under reflux at 78°C for 2 h, as previously described [11], with the yield being 14% (w/w). The extract was further fractionated using silica gel chromatography [12]. HCF1 was obtained at a yield of 1.14% (w/w). The extract was dried by evaporating the solvent under reduced pressure at 50°C, and the dried extract was stored at −20°C prior to use.

### 2.2. Chemicals

Anti-*β*-actin antibody, BSS (CAS 83-46-5), protease inhibitor cocktail, and phosphatase inhibitor cocktail 3 were purchased from Sigma (St Louis, MO). Anti-AMPK*α*1/2 antibody, anti-p-AMPK*α*1/2 antibody, anti-PGC-1 (H-300) antibody, anti-UCP3 (E-18) antibody, 6-[4-(2-piperidin-1-yl-ethoxy)-phenyl)]-3-pyridin-4-yl-pyrrazolo[1,5-a]-pyrimidine (CC), and 6-ketocholestanol (kCh) were purchased from Santa Cruz Biotechnology (Santa Cruz, CA, USA). Anti-COX antibody was purchased from Cell Signaling (Danvers, MA). Anti-lamin B1 antibody was purchased from Abcam (Cambridge, MA). Bio-Rad assay reagent was purchased from Bio-Rad Laboratories (Richmond, CA, USA). All other chemicals were purchased from Sigma (St. Louis, MO, USA).

### 2.3. Cell Culture

C2C12, a C3H mouse skeletal muscle-derived myoblast culture [13] from the American Type Culture Collection (ATCC), was kindly provided by Professor Zhenguo WU (Division of Life Science, HKUST). The cell line was cultured in Dulbecco's modified Eagle's medium (DMEM) (Gibco BRL Life Technologies, Grand Island, NY), supplemented with 10% (v/v) fetal bovine serum (FBS), 100 IU/mL penicillin and 100 *μ*g/mL of streptomycin, and 17 mM NaHCO_3_. The differentiation of C2C12 myoblasts to C2C12 myotubes was induced by substituting complete culture medium with differentiation medium containing 2% (v/v) horse serum (HS) instead of 10% (v/v) FBS. Cells were grown under an atmosphere of 5% (v/v) CO_2_ in air at 37°C.

### 2.4. Biochemical Analyses in C2C12 Myotubes

#### 2.4.1. MMP

Mitochondrial membrane potential was measured using a fluorescent cationic dye, 5,5′,6,6′-tetrachloro-1,1′,3,3′-tetraethylbenzimidazolyl-carbocyanine iodide (JC-1) [14]. Cells were cultured at a density of 1.0 × 10^4^ cells in 96-well microtiter plates. After stable attachment, cells were prestained with 20 *μ*M JC-1 (dissolved in culture medium) at 37°C for 10 min. Following prestaining, cells were washed twice with Hank's balanced salt solution (HBSS), supplemented with 0.1% bovine serum albumin (BSA). The measurement of MMP was initiated by the addition of HCF1/BSS-containing medium. The amount of J-aggregate of JC-1 molecules, which forms in the inner membrane of mitochondria of high membrane potential, was determined by monitoring JC-1 red fluorescence (Ex 485 nm/Em 580 nm) at 37°C for 1 h. MMP was estimated by subtracting the fluorescence intensities of blank samples (i.e., containing medium only) from the fluorescence intensities of tested samples. MMP values of HCF1/BSS-incubated cells were normalized with the respective non-HCF1/BSS-incubated control values and expressed as percent control.

#### 2.4.2. Adenosine Monophosphate-Dependent Protein Kinase (AMPK) Activation

C2C12 myotubes were seeded (1.5 × 10^5^ cells) in 6-well microtiter plates. After stable attachment, cells were preincubated with HCF1/BSS-containing medium for 8 h. The preincubated cells were then lysed with 300 *μ*L of sodium dodecyl sulfate- (SDS-) lysis buffer (20 mM Tris-Cl, 2 mM ethylenediaminetetraacetic acid (EDTA), 3 mM ethylene glycol tetraacetic acid (EGTA), 1% (w/v) Triton X-100, 10% (w/v) glycerol, 5% SDS, 1 mM dithiothreitol (DTT), protease inhibitor cocktail, and phosphatase inhibitor cocktail 3, pH 7.5). The lysate was further centrifuged at 12000 ×g at 4°C for 10 min to remove cell debris. The extent of AMPK activation was assessed from the relative ratio of phospho-AMPK*α* (p-AMPK) and AMPK*α* by Western blot analysis using anti-p-AMPK*α*1/2 antibody and anti-AMPK*α*1/2 antibody following SDS-PAGE analysis, with a separating gel of 10% (v/v) acrylamide. Cell lysate (20 *μ*g) was loaded and *β*-actin was used as a reference marker. The immune-stained protein bands were analyzed by densitometry (Quantiscan, Biosoft), and the amounts (arbitrary units) of p-AMPK*α* and AMPK*α* were normalized with reference to the *β*-actin level (arbitrary units) in the sample.

#### 2.4.3. PGC-1 Nuclear Translocation

C2C12 myotubes were cultured at a density of 2.0 × 10^6^ cells in 100 mm culture plates. After 8 h HCF1/BSS preincubation cells were trypsinized and incubated with 1 mL ice-cold hypotonic buffer (10 mM HEPES, 1.5 mM KCl, and 1.5 mM MgCl_2_, pH 7.9) at 4°C for 10 min. Following incubation, cells were collected by centrifugation at 500 ×g at 4°C for 10 min. The collected cells were resuspended in 300 *μ*L hypotonic buffer, supplemented with 0.5 mM DTT, 0.1% (w/v) nonyl phenoxypolyethoxylethanol (NP-40), and protease inhibitor cocktail, to facilitate the extraction of soluble cytosolic proteins. The resultant lysate was centrifuged at 12000 ×g at 4°C for 1 min to yield cytosolic (supernatant) and nuclear (pellet) fractions. The isolation of nuclear proteins was achieved by incubating the pellet with 25 *μ*L ice-cold hypertonic buffer (20 mM HEPES, 25% (w/v) glycerol, 400 mM NaCl, 1.5 mM MgCl_2_, 0.2 mM EDTA, 0.5 mM DTT, and protease inhibitor cocktail, pH 7.9) at 4°C for 30 min followed by centrifugation at 12000 ×g at 4°C for 5 min. The extent of PGC-1 nuclear translocation was estimated from the relative ratio of the PGC-1 level in cytosolic and nuclear fractions using Western blot analysis following SDS-PAGE analysis, as described above. Both cytosolic and nuclear fractions (20 *μ*g) were loaded, with *β*-actin and lamin B1 being used as cytosolic and nuclear reference markers, respectively. The amounts (arbitrary units) of PGC-1 were normalized with reference to *β*-actin and lamin B1 levels, respectively, (arbitrary units) in the sample.

#### 2.4.4. Mitochondrial Uncoupling Protein 3 (UCP3) and Cytochrome c Oxidase (COX) Expression

C2C12 myotubes were seeded (2.0 × 10^6^ cells) in 100 mm culture plates. After stable attachment, cells were preincubated with HCF1/BSS-containing medium at 37°C for 48 h. After removal of HCF1/BSS-containing medium, cells were trypsinized and lysed by the addition of 300 *μ*L SDS-lysis buffer. Cell lysate was centrifuged at 12000 ×g at 4°C for 10 min to remove cell debris. UCP3 and COX levels in the supernatant were determined by Western blot analysis using anti-UCP3 (E-18) antibody and anti-COX antibody following SDS-PAGE analysis as described above. Cell lysate (50 *μ*g) was loaded and *β*-actin was used as a reference marker. The amounts (arbitrary units) of UCP3 and COX were normalized with reference to the *β*-actin level (arbitrary units) in the sample.

### 2.5. Animal Care

ICR mice (8 weeks; 30 to 35 g for males and 25 to 30 g for females) were maintained under a 12 h dark/light cycle in an air/humidity-controlled environment at about 22°C and allowed food and water* ad libitum* in the Animal and Plant Care Facilities (APCF) at Hong Kong University of Science and Technology (HKUST). All experimental procedures were approved by the Research Practice Committee (HKUST) (animal protocol approval number 2013049; approval date: 25 September 2013; experiment duration: 3 years).

### 2.6. Treatment Protocol

To examine the effect of 6-ketocholestanol (kCh) on HCF1-induced weight reduction in HFD-fed mice, animals were randomly assigned to 6 groups, with 10–15 mice in each: (1) normal diet (ND, 13% energy derived from fat, purchased from LabDiet, product number 5010) control; (2) ND + kCh (at a daily dose of 9 mg/kg); (3) HFD (60% energy derived from fat; purchased from Research Diet Inc., product number D12492) control; (4) HFD + HCF1 (at a daily dose of 45 mg/kg); (5) HFD + kCh; and (6) HFD + HCF1 + kCh. HCF1, at a daily dose of 45 mg/kg, was shown to produce significant weight reduction in male mice [4], while kCh, at a daily dose of 9 mg/kg, was found to abrogate the HCF1-induced uncoupling effect in mitochondria isolated from mouse skeletal muscle (data not shown). Animals were intragastrically administered kCh, HCF1, or HCF1/kCh, 5 days per week, for 8 weeks (i.e., for a total of 40 doses). Control mice received the vehicle (olive oil) only. Body weights and food consumption of the mice were monitored weekly during the course of the experiment. The changes in body weight were quantified by calculating the area under the curve (AUC) of a graph plotting percent initial body weight against time (week 1 to 8) and expressed in arbitrary units. The mice were sacrificed by cervical dislocation 24 h after the last dosing (with kCh, HCF1, or HCF1/kCh) following overnight fasting. Samples of gastrocnemius muscle were excised for the measurement of mitochondrial respiration. Fat pads (gonadal, retroperitoneal, and mesenteric fat) were dissected and weighed. The ratio of a particular fat pad weight to body weight was estimated and expressed as “fat pad index”.

### 2.7. Biochemical Analyses in Mice

#### 2.7.1. Sample Preparations

Minced gastrocnemius muscle tissues were mixed with 10 mL collagenase solution (0.075% (w/v)) in buffer (100 mM KCl, 50 mM MOPS, pH 7.2), and the mixtures were incubated at 4°C for 20 min. The digested tissue mixtures were centrifuged at 600 ×g at 4°C for 20 min. The supernatant was removed; the digested tissues were mixed with 20 mL of ice-cold homogenizing buffer (50 mM sucrose, 200 mM mannitol, 5 mM KH_2_PO_4_, 1 mM EGTA, and 5 mM MOPS, pH 7.2) and were homogenized on ice with a Teflon-glass homogenizer at 4000 rpm for 25 to 30 complete strokes. The homogenates were then centrifuged at 600 ×g at 4°C for 10 min, yielding a nucleus-free fraction (supernatant). Mitochondrial pellets were prepared from muscle homogenates by centrifugation at 9200 ×g at 4°C for 30 min. Mitochondrial fractions were obtained by suspending the pellets in a buffer containing 125 mM KCl, 10 mM Tris-Base, 2 mM KH_2_PO_4_, 0.5 mM EGTA, and 20 mM MOPS, pH 7.5 [15].

#### 2.7.2. Mitochondrial Respiration

Respiration rates in mitochondria isolated from mouse skeletal muscle were determined as described by Wong and Ko [12]. In brief, mitochondrial respiration was measured polarographically using a Clark-type electrode (Hansatech Instruments, Norfolk) at 30°C. Mitochondrial fractions (1 mg protein/mL) were incubated in the respiration buffer (30 mM KCl, 6 mM MgCl_2_, 75 mM sucrose, 1 mM EDTA, and 20 mM KH_2_PO_4_, pH 7.0). Substrate solution containing 15 mM sodium pyruvate and 5 mM sodium malate was added. After equilibration, state 3 respiration was initiated by the addition of ADP (final concentration 0.6 mM). When all of the added ADP was used up for ATP generation, oligomycin was added to induce state 4 respiration. The respiratory control ratio (RCR) was estimated by calculating the ratio of state 3 to state 4 respiration rates [16].

#### 2.7.3. Mitochondrial UCP3 Expression in Mouse Skeletal Muscle

Mitochondrial UCP3 levels were estimated by Western blot analysis using anti-UCP3 (E-18) antibody following SDS-PAGE analysis of the mitochondrial fraction, using a separating gel of 10% acrylamide. Mitochondrial fractions isolated from mouse skeletal muscle (~60 *μ*g) were applied to the gel and cytochrome c oxidase (COX) was used as a reference marker. The immunostained protein bands were analyzed by densitometry and the amounts (arbitrary units) of UCP3 normalized with reference to the COX level (arbitrary units) in the sample.

### 2.8. Protein Assay

Protein concentrations were determined using a Bio-Rad protein assay kit. An aliquot (10 *μ*L) of mitochondrial/cell lysate sample was diluted with 40 *μ*L of water. An aliquot (10 *μ*L) of diluted sample was then added to a well of a 96-well microtiter plate, followed by the addition of 200 *μ*L of diluted Bio-Rad assay reagent (5-fold dilution). The mixture was allowed to stand at room temperature for 5 minutes and the absorbance of the reaction mixture at 570 nm was then measured. Protein concentration was determined from a calibration curve using bovine serum albumin (BSA) as standard.

### 2.9. Statistical Analysis

All data were expressed as mean ± standard error of the mean (SEM), unless otherwise specified. Data were analyzed by one-way analysis of variance (one-way ANOVA), unless otherwise specified, and intergroup differences were detected using the Tukey range test, with *P* < 0.05.

## 3. Results

### 3.1. Effects of HCF1/BSS on MMP in C2C12 Myotubes

**Figure 1:**
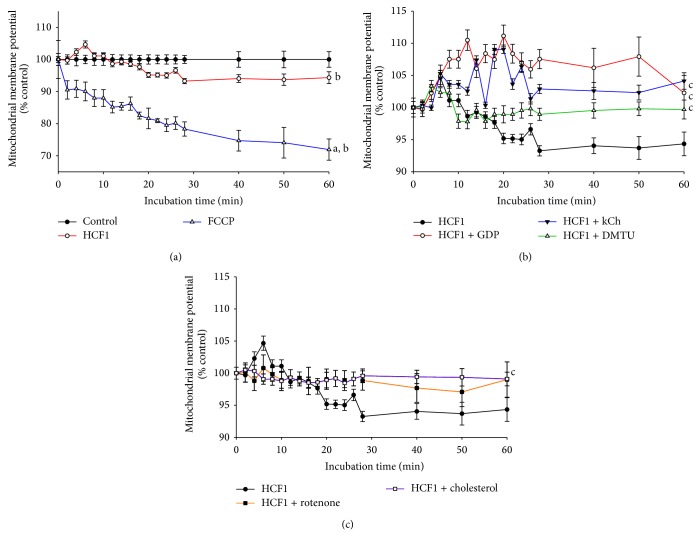
Time-course of HCF1-induced changes in mitochondrial membrane potential in C2C12 myotubes. Mitochondrial membrane potential was measured as described in Materials and Methods section. Data were analyzed by mixed-design analysis of variance and are expressed as percent control with respect to the non-HCF1 incubated time-match controls. Values given are means ± SEM, with *n* = 6; ^a^significantly different from non-drug preincubated time-match controls (0 to 10 min); ^b^significantly different from non-drug preincubated time-match controls (11 to 60 min); ^c^significantly different from HCF1 preincubated time-match controls (11 to 60 min) (*P* < 0.05).

**Figure 2:**
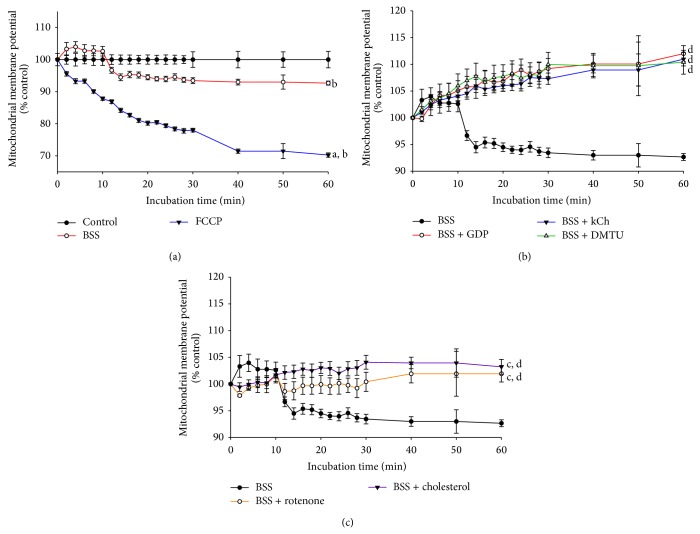
Effects of BSS on mitochondrial membrane potential in C2C12 myotubes. Cells were prestained with JC-1 at 37°C for 10 min. The measurement of mitochondrial membrane potential was initiated immediately after the addition of the test compounds. Data were analyzed by mixed-design analysis of variance and are expressed as percent control with respect to the non-HCF1 incubated time-match controls. Values given are means ± SEM, with *n* = 6; ^a^significantly different from non-drug preincubated time-match controls (0 to 10 min); ^b^significantly different from non-drug preincubated time-match controls (11 to 60 min); ^c^significantly different from HCF1 preincubated time-match controls (0 to 10 min); ^d^significantly different from HCF1 preincubated time-match controls (11 to 60 min) (*P* < 0.05).

Incubation of the chemical uncoupler, carbonyl cyanide 4-(trifluoromethoxy) phenylhydrazone (FCCP), at a concentration of 1 *μ*M, caused a time-dependent reduction in MMP, with the extent of reduction being 29% at the end of the measurement (Figures 1 and 2). In contrast, HCF1 and BSS, at concentrations of 30 ng/mL and 10 *μ*M, increased the MMP by 5 and 4%, respectively, in the first 10 min of HCF1/BSS incubation (Figures 1 and 2). The initial HCF1/BSS-induced increases were followed by gradual decreases in MMP to 95 and 93%, respectively, of the respective initial value at the end the measurement (60 min HCF1/BSS incubation) (Figures 1 and 2). The HCF1/BSS-induced changes in MMP were characterized by the use of specific inhibitors. Rotenone (0.1 nM), a mitochondrial complex I inhibitor, and cholesterol (0.3 *μ*M), an enhancer of membrane rigidity, suppressed the HCF1/BSS-induced changes in MMP throughout the course of the measurement (Figures 1 and 2). On the other hand, coincubation with a specific inhibitor of UCPs, GDP, a chemical recoupler, 6-ketocholestanol (kCh), or an antioxidant, dimethylthiourea (DMTU), diminished the HCF1-induced reduction in MMP at the later time of HCF1 incubation (Figure 1). GDP, kCh, and DMTU also diminished the BSS-induced reduction in MMP at the later time of BSS incubation, with the MMP value being 110%, when compared with the respective time-matched control (Figure 2).

### 3.2. Effect of HCF1/BSS on AMPK Activation in C2C12 Myotubes

**Figure 3:**
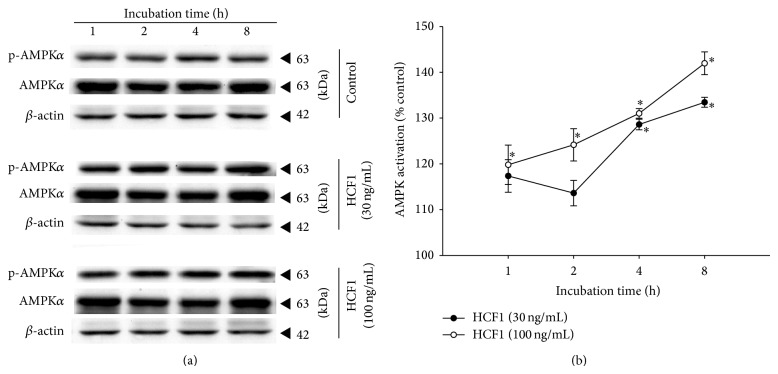
Time-course of HCF1-induced changes in AMPK activation in C2C12 myotubes. Cells were preincubated with HCF1 for increasing periods of time at 37°C. The extent of AMPK activation was assessed as described in Materials and Methods section. Data are expressed as percent control with respect to non-HCF1 preincubated time-match controls. Values given are means ± SEM, with *n* = 4. ^*^Significantly different from non-drug preincubated time-match controls (*P* < 0.05).

**Figure 4:**
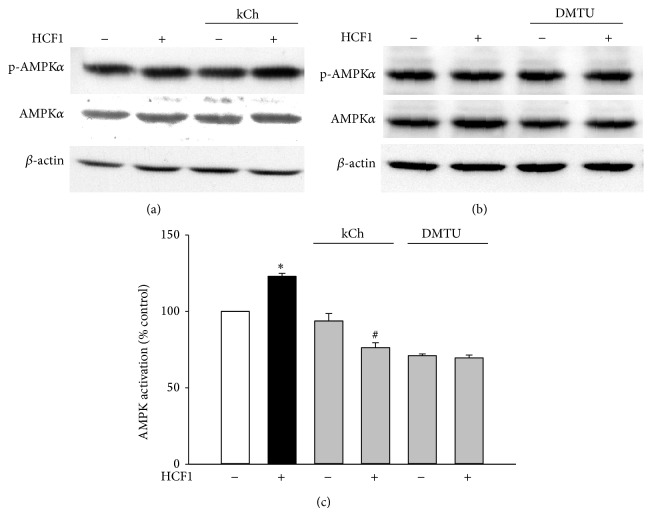
Effects of kCh and DMTU on HCF1-induced AMPK activation in C2C12 myotubes. Data are expressed as percent control with respect to non-HCF1 preincubated controls. Values given are means ± SEM, with *n* = 4. ^*^Significantly different from the non-HCF1 preincubated control group; ^#^significantly different from the kCh preincubated, HCF1 unpreincubated control group (*P* < 0.05).

**Figure 5:**
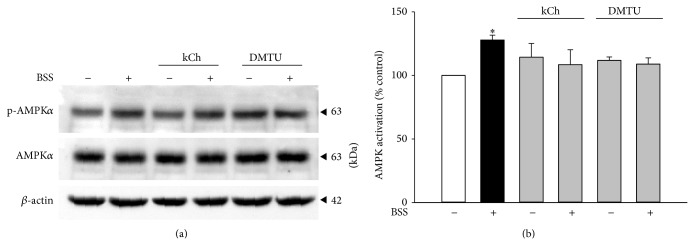
Effects of BSS on AMPK activation in C2C12 myotubes. Data are expressed as percent control with respect to non-BSS preincubated controls. Values given are means ± SEM, with *n* = 4. ^*^Significantly different from the non-drug preincubated control group (*P* < 0.05).

The effect of HCF1 on AMPK was examined by measuring the ratio of phosphorylated AMPK*α* (p-AMPK*α*) to total AMPK*α* (AMPK*α*), which indicates the extent of AMPK activation. Incubation with HCF1, at concentrations of 30 and 100 ng/mL, produced time-dependent increases in AMPK activation, with the extent of these increases being 32 and 43% at 8 h of HCF1 incubation, respectively, when compared with the respective time-matched control (Figure 3). In parallel to HCF1, preincubation with BSS (10 *μ*M) significantly increased the extent of AMPK activation by 25%, when compared with the unpreincubated control (Figure 5). The HCF1/BSS-induced AMPK activation in C2C12 myotubes was completely abrogated by coincubation with either kCh (a recoupler) or DMTU (Figures 4 and 5, Tables  1 and 2 of Supplementary Material available online at http://dx.doi.org/10.1155/2015/142059).

### 3.3. Effect of HCF1/BSS on PGC-1 Nuclear Translocation in C2C12 Myotubes

**Figure 6:**
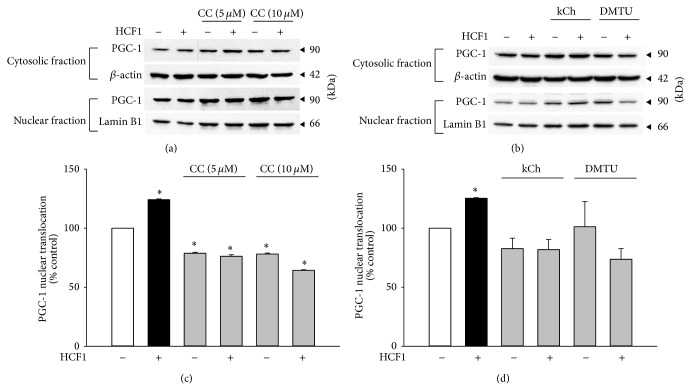
Effects of HCF1 on PGC-1 nuclear translocation in C2C12 myotubes. Cells were preincubated with HCF1 for 8 h. The extent of PGC-1 nuclear translocation was determined by the partition of PGC-1 between cytosolic and nuclear fractions, as described in Materials and Methods section. Data are expressed as percent control with respect to non-HCF1 preincubated controls. Values given are means ± SEM, with *n* = 4. ^*^Significantly different from the unpreincubated control group (*P* < 0.05).

**Figure 7:**
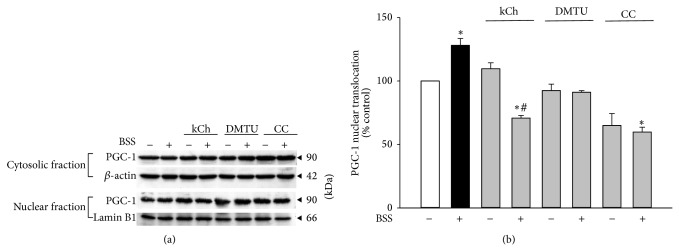
Effects of BSS on PGC-1 nuclear translocation in C2C12 myotubes. Cells were preincubated with BSS for 8 h. The extent of PGC-1 nuclear translocation was determined by Western blot analysis. Data are expressed as percent control with respect to the corresponding non-BSS preincubated control. Values given are means ± SEM, with *n* = 4. ^*^Significantly different from the unpreincubated control group; ^#^significantly different from kCh preincubated, HCF1 unpreincubated control group (*P* < 0.05).

The activation of PGC-1 can be indirectly measured by the extent of PGC-1 nuclear translocation. Preincubation with HCF1 and BSS significantly increased PGC-1 nuclear translocation by 25 and 28%, respectively, as evidenced by elevated ratios of PGC-1 in nucleus to cytoplasm (Figures 6 and 7). A specific inhibitor of AMPK, 6-[4-(2-piperidin-1-yl-ethoxy)-phenyl)]-3-pyridin-4-yl-pyrrazolo[1,5-a]-pyrimidine (CC), at a concentration of 10 *μ*M, inhibited the HCF1/BSS-induced increase in PGC-1 nuclear translocation by 156 and 235%, respectively, in C2C12 myotubes (Tables  1 and  2). The HCF1/BSS-induced nuclear translocation of PGC-1 was also prevented by coincubation with either kCh or DMTU (Figures 6 and 7, Tables  1 and  2 of Supplementary Material).

### 3.4. Effect of HCF1/BSS on Mitochondrial UCP3 and Cytochrome c Oxidase (COX) Expression in C2C12 Myotubes

**Figure 8:**
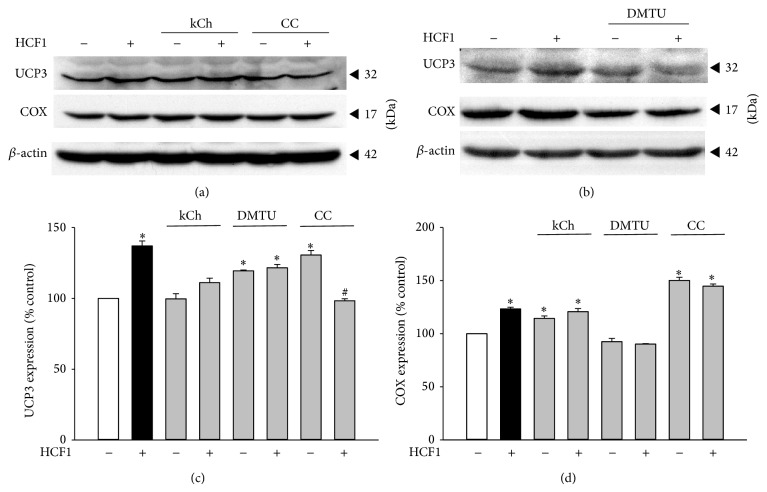
Effects of HCF1 on mitochondrial UCP3 and COX expression in C2C12 myotubes. Cells were preincubated with HCF1 for 48 h. The levels of mitochondrial UCP3 and COX were measured by Western blot analysis. Data are expressed as percent control with respect to non-drug preincubated controls. Values given were means ± SEM, with *n* = 4. ^*^Significantly different from unpreincubated control groups; ^#^significantly different from the CC preincubated, HCF1 unpreincubated control group (*P* < 0.05).

**Figure 9:**
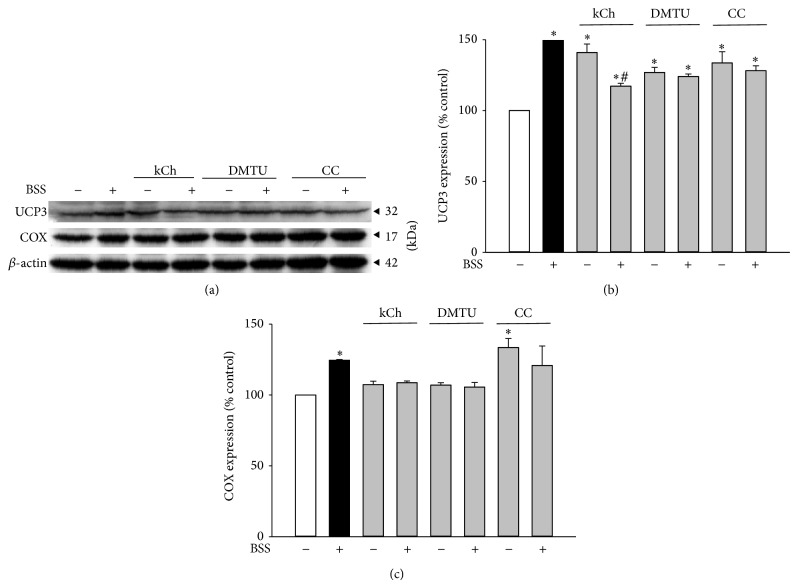
Effects of BSS on mitochondrial UCP3 and COX expression in C2C12 myotubes. The levels of mitochondrial UCP3 and COX were measured as described in Materials and Methods. Data are expressed as percent control with respect to the corresponding unpreincubated control. Values given were means ± SEM, with *n* = 4. ^*^Significantly different from the non-drug preincubated control group (*P* < 0.05).

To determine the significance of HCF1/BSS-induced activation in the AMPK/PGC-1 pathway, the effects of HCF1/BSS on mitochondrial uncoupling and biogenesis, as assessed by levels of UCP3 and COX (a reliable indicator of mitochondrial mass in cells), respectively, were investigated. Preincubation with HCF1/BSS significantly increased mitochondrial UCP3 and COX levels, with the extent of these increases being 37 and 23% (in HCF1-incubated myotubes) (Figure 8) and 52 and 24% (in BSS-incubated myotubes) (Figure 9), respectively, when compared with the respective unpreincubated control. The HCF1/BSS-induced elevations in UCP3 and COX levels were completely prevented by coincubation of HCF1 with kCh, DMTU, or CC (Tables  1 and  2 of Supplementary Material). It should be noted that the concentrations of kCh and CC used in this experiment were adjusted to 20 and 1 *μ*M, respectively, to avoid the cytotoxic effects of kCh and CC on C2C12 myotubes (unpublished observation).

### 3.5. The Role of Mitochondrial Uncoupling in HCF1-Induced Weight Reduction in HFD-Induced Obese Mice

The possible role of mitochondrial uncoupling in HCF1-induced weight reduction was further examined by the use of the chemical recoupler, kCh (Table  3 of Supplementary Material). HCF1 induced mitochondrial uncoupling in mouse skeletal muscle, as evidenced by the reduction in the mitochondrial respiratory control ratio (RCR) and the increase in mitochondrial UCP3 expression, in both ND- and HFD-fed mice. kCh* per se* (at a daily dose of 9 mg/kg) significantly increased mitochondrial RCR by 23% in ND-fed mice (but not in HFD-fed mice). The HCF1-induced mitochondrial uncoupling in HFD-fed mice was completely inhibited by kCh cotreatment. HCF1 produced weight reduction in both ND- and HFD-fed mice. This effect was associated with the suppression of the HFD-induced increase in visceral fat index. In contrast to HCF1, kCh produced no detectable effect on weight gain in ND- and HFD animals during the 8-week course of the experiment. The coadministration of kCh with HCF1 abolished the HCF1-induced suppressive effects on HFD-induced increase in body weight and visceral fat index in mice (Table  3 of Supplementary Material).

## 4. Discussion

HCF1, a semipurified fraction of Cistanches Herba, was shown to produce weight reduction in HFD-induced obese mice. The weight reduction was associated with the induction of mitochondrial uncoupling and changes in energy metabolic enzyme activities in mouse skeletal muscle [4]. HCF1 and its active component, BSS, have been shown to decrease mitochondrial coupling efficiency secondary to the production of mitochondrial reactive oxygen species (ROS) in H9c2 cardiomyocytes and rat hearts [17]. In the present study, to elucidate the biochemical mechanism underlying the HCF1-induced mitochondrial uncoupling in mouse skeletal muscle, the effects of HCF1 and BSS on MMP in C2C12 myotubes were investigated.

MMP is generated in the IMM as a consequence of proton pumping by the mitochondrial electron transport chain. Changes in MMP reflect an interplay of mitochondrial electron transport and the dissipation of the proton gradient by various processes, such as ATP synthase-mediated ATP generation, proton leak via IMM, or an UCP-mediated mitochondrial uncoupling [18, 19]. HCF1/BSS incubation caused a transient elevation in MMP in C2C12 myotubes. The ability of rotenone, a mitochondrial respiratory complex I inhibitor, to suppress the HCF1/BSS-induced elevation in MMP suggests that this process was likely mediated by an increase in mitochondrial electron transport. This is consistent with our previous finding that HCF1/BSS increased mitochondrial state 3 respiration in H9c2 cardiomyocytes and rat hearts [17]. In addition, Shi et al. (2013) [20] reported that the incorporation of BSS into mitochondrial membranes specifically fluidizes the IMM, with consequent increases in mitochondrial membrane potential and mitochondrial ATP content. Our finding that the coincubation with cholesterol, an enhancer of membrane rigidity [21], abolished the HCF1/BSS-induced increase in MMP suggests the involvement of an increase in mitochondrial membrane fluidity in the HCF1/BSS-induced increase in MMP. The ability of rotenone and GDP to abrogate the HCF1/BSS-induced decrease in MMP observable at the later period of HCF1/BSS incubation suggests the induction of an UCP3-mediated mitochondrial uncoupling secondary to the stimulation in mitochondrial electron transport. DMTU, an antioxidant, prevented HCF1/BSS-induced mitochondrial uncoupling, suggesting a redox-sensitive activation of UCP3 in C2C12 myotubes, which is likely mediated by the glutaredoxin 2 (GRx2) induced S-deglutathionylation of UCP3. GRx2 is a matrix oxidoreductase, which modulates UCPs activities via reversible S-glutathionylation in response to the changes in the cellular redox environment [18, 22, 23]. Taken together, our findings suggest that HCF1/BSS may fluidize the IMM, resulting in the stimulation of mitochondrial electron transport, with a resultant increase in mitochondrial ROS production. The elevated mitochondrial ROS, in turn, would trigger a redox-sensitive activation of mitochondrial uncoupling in C2C12 myotubes.

In view of the HCF1-induced changes in energy metabolic enzyme activities in the skeletal muscle of HFD-fed mice, the effects of HCF1/BSS on the AMPK/PGC-1 signaling pathway, which participates actively in the control of cellular energy homeostasis [7, 8], was examined. The finding that HCF1/BSS induced a DMTU-sensitive activation of AMPK indicated the ability of HCF1/BSS to trigger a redox-sensitive AMPK activation in C2C12 myotubes. Recently, AMPK has been reported to be activated by ROS as well as reactive nitrogen species (RNS), with a resulting enhancement of cell viability under conditions of oxidative stress. The exposure of AMPK to oxidative stress leads to the oxidation of cysteine residues of the *α*- and *β*-subunit, with resultant allosteric rearrangement of the heterotrimeric complex and thereby the activation of kinase activity [24, 25]. In addition, the S-glutathionylation of AMPK, as catalyzed by glutathione transferase (GST) and GRx, following H_2_O_2_ exposure was found to stimulate its kinase activity [26]. Taken together, our findings support the notion that HCF1/BSS can activate AMPK by way of mitochondrial ROS production. The HCF1/BSS-induced activation of AMPK was also found to be associated with an increased nuclear translocation of PGC-1. In this regard, AMPK can posttranslationally activate PGC-1 by phosphorylation of the Thr177/Ser538 of PGC-1 and thereby facilitate the deacetylation of PGC-1 by sirtuin 1 (SIRT1), which is a critical step for PGC-1*α* activation [7]. The activated PGC-1*α*, in turn, interacts with nuclear receptors such as peroxisome proliferator-activated receptor *γ* (PPAR*γ*), nuclear respiratory factors (NRFs), and myocyte enhancer factor 2C (MEF2C), with resultant nuclear translocation and coactivation of gene expression [27–29]. The inhibition of HCF1/BSS-induced PGC-1 nuclear translocation by DMTU and the AMPK inhibitor, CC, indicates that the PGC-1 activation is an event secondary to ROS-induced AMPK activation.

By coactivating multiple transcription factors, AMPK/PGC-1 exerts a wide spectrum of actions in controlling gene expression related to muscle fiber type switching, uptake and utilization of fuel molecules, and mitochondrial metabolism, all of which provide an orchestrated network to regulate cellular energy metabolism and thereby improve overall metabolic fitness [30–32]. In this connection, our findings suggest the possible involvement of AMPK/PGC-1 in the beneficial effect produced by HCF1 on glucose and fatty acid metabolism in both ND-fed and HFD-fed mice [4]. HCF1/BSS incubation also increased UCP3 expression and stimulated mitochondrial biogenesis via the redox-sensitive activation of AMPK/PGC-1 in C2C12 myotubes. The increased expression of UCP3 in response to HCF1/BSS, which was also observed in mouse skeletal muscle of ND- and HFD-fed mice, suggests the involvement of the AMPK/PGC-1 pathway in the upregulation of UCP3 expression in mouse skeletal muscle. Furthermore, the HCF1/BSS-induced increase in mitochondrial biogenesis suggests an enhanced mitochondrial oxidative capacity with a resultant improvement in metabolic fitness. Given that the redox-sensitive activation of the AMPK/PGC-1 signaling pathway is crucial in defending against oxidant injury in various tissues [24, 33, 34], HCF1/BSS may offer an alternative approach to ablating obesity-related oxidative stress.

The exploration of pharmacological interventions aimed at ameliorating obesity has been an area of intensive research. Currently, most of the slimming agents are mainly food substitutes, appetite suppressants, and functional compounds that stimulate the sympathetic nervous system (SNS) [35]. The finding that HCF1 produced weight reduction in HFD-induced obesity via the redox-sensitive induction of mitochondrial uncoupling offers an alternative approach to weight control. Mitochondrial uncoupling has long been proven to be effective in inducing weight loss in animals and humans [36]. The findings from our previous and present studies unequivocally attest to the ability of HCF1 to produce weight reduction via the induction of mitochondrial uncoupling.

## 5. Conclusion

A Cistanches Herba fraction (HCF1)/BSS causes the redox-sensitive induction of mitochondrial uncoupling and activation of AMPK/PGC-1 in C2C12 myotubes. HCF1 treatment may increase in the bodily energy consumption, particularly in skeletal muscle, with resultant reductions in body weight and adiposity in HFD-fed mice.

## Supplementary Material

Supplementary Table 1 and 2: The HCF1/BSS-induced AMPK activation in C2C12 myotubes was completely abrogated by co-incubation with either kCh or DMTU. A specific inhibitor of AMPK, CC, at a concentration of 10 µM, inhibited the HCF1/BSS-induced increase in PGC-1 nuclear translocation in C2C12 myotubes. The HCF1/BSS-induced nuclear translocation of PGC-1 was also prevented by co-incubation with either kCh or DMTU. The HCF1/BSS-induced elevations in UCP3 and COX levels were completely prevented by co-incubation of HCF1with kCh, DMTU or CC.
Supplementary Table 3: The possible role of mitochondrial uncoupling in HCF1-induced weight reduction was examined by the use of the chemical recoupler, kCh. HCF1 induced mitochondrial uncoupling in mouse skeletal muscle, as evidenced by the reduction in the mitochondrial RCR and the increase in mitochondrial UCP3 expression, in both ND- and HFD-fed mice. kCh per se significantly increased mitochondrial RCR in ND-fed mice. The HCF1-induced mitochondrial uncoupling in HFD-fed mice was completely inhibited by kCh co-treatment. HCF1 produced weight reduction in both ND- and HFD-fed mice. This effect was associated with the suppression of the HFD-induced increase in visceral fat index. In contrast to HCF1, kCh produced no detectable effect on weight gain in ND- and HFD-animals during the 8-week course of the experiment. The co-administration of kCh with HCF1 abolished the HCF1-induced suppressive effects on HFD-induced increase in body weight and visceral fat index in mice.
