# Supplementary material for: A Cistanches Herba Fraction/β-Sitosterol Causes a Redox-Sensitive Induction of Mitochondrial Uncoupling and Activation of Adenosine Monophosphate-Dependent Protein Kinase/Peroxisome Proliferator-Activated Receptor γ Coactivator-1 in C2C12 Myotubes: A Possible Mechanism Underlying the Weight Reduction Effect
Source: Evid Based Complement Alternat Med. 2015 Feb 3;2015:142059. doi: 10.1155/2015/142059 (PMC4332982; doi:10.1155/2015/142059)
Supplement: Supplementary file 1 — Supplementary Table 1 and 2: The HCF1/BSS-induced AMPK activation in C2C12 myotubes was completely abrogated by co-incubation with either kCh or DMTU. A specific inhibitor of AMPK, CC, at a concentration of 10 µM, inhibited the HCF1/BSS-induced increase in PGC-1 nuclear translocation in C2C12 myotubes. The HCF1/BSS-induced nuclear translocation of PGC-1 was also prevented by co-incubation with either kCh or DMTU. The HCF1/BSS-induced elevations in UCP3 and COX levels were completely prevented by co-incubation of HCF1with kCh, DMTU or CC. Supplementary Table 3: The possible role of mitochondrial uncoupling in HCF1-induced weight reduction was examined by the use of the chemical recoupler, kCh. HCF1 induced mitochondrial uncoupling in mouse skeletal muscle, as evidenced by the reduction in the mitochondrial RCR and the increase in mitochondrial UCP3 expression, in both ND- and HFD-fed mice. kCh per se significantly increased mitochondrial RCR in ND-fed mice. The HCF1-induced mitochondrial uncoupling in HFD-fed mice was completely inhibited by kCh co-treatment. HCF1 produced weight reduction in both ND- and HFD-fed mice. This effect was associated with the suppression of the HFD-induced increase in visceral fat index. In contrast to HCF1, kCh produced no detectable effect on weight gain in ND- and HFD-animals during the 8-week course of the experiment. The co-administration of kCh with HCF1 abolished the HCF1-induced suppressive effects on HFD-induced increase in body weight and visceral fat index in mice. [file 142059.f1.pdf]

Supplementary materials

Table 1 The effects of kCh, DMTU and CC on the HCF1-induced AMPK/PGC-1 activation in C2C12 myotubes

(% control)	HCF1	HCF1 + kCh	HCF1 + DMTU	HCF1 + CC
AMPK activation	$25.8^{*} \pm 2.0$	$-17.5^{\#} \pm 4.9$	$2.8^{\#} \pm 2.0$	- -
PGC-1 nuclear translocation	$25.3^{*} \pm 0.6$	$-0.9^{\#} \pm 8.6$	$-27.5^{\#} \pm 9.1$	$-13.7^{\#} \pm 1.4$
Mitochondrial UCP3 expression	$36.9^{*} \pm 5.0$	$11.5^{\#} \pm 5.5$	$2.1^{\#} \pm 2.4$	$-32.3^{\#} \pm 2.5$
Mitochondrial COX expression	$23.2^{*} \pm 2.1$	$6.3^{\#} \pm 4.3$	$-2.2^{\#} \pm 3.2$	$-5.5^{\#} \pm 1.4$

Data are expressed as percent control with respect to the unpre-incubated control. Values given are means ± SEM, with n = 4.

* Significantly different from the unpre-incubated control; # significantly different from the HCF1-pre-incubated control

Table 2 The effects of kCh, DMTU and CC on the BSS-induced AMPK/PGC-1 activation in C2C12 myotubes

(% control)	BSS	BSS + kCh	BSS + DMTU	BSS + CC
AMPK activation	$25.4^{*} \pm 5.4$	$-5.8^{\#} \pm 11.7$	$-15.4^{\#} \pm 8.9$	- -
PGC-1 nuclear translocation	$28.1^{*} \pm 9.2$	$-38.8^{\#} \pm 3.4$	$-2.7^{\#} \pm 4.5$	$-5.1^{\#} \pm 6.5$
Mitochondrial UCP3 expression	$52.6^{*} \pm 1.6$	$-23.8^{\#} \pm 2.5$	$2.9^{\#} \pm 1.4$	$-5.5^{\#} \pm 5.9$
Mitochondrial COX expression	$23.5^{*} \pm 1.2$	$1.3^{\#} \pm 2.1$	$-1.4^{\#} \pm 3.2$	$-12.7^{\#} \pm 11.1$

Data are expressed as percent control with respect to the unpre-incubated control. Values given are means ± SEM, with n = 4.

* Significantly different from the unpre-incubated control; # significantly different from the BSS-pre-incubated control

Table 3 The effect of HCF1 and kCh on HFD-induced obese mice

	ND		HFD			
% ND control	HCF1	kCh	Control	HCF1	kCh	HCF1/kCh
Mitochondrial RCR	68.8* ± 3.6	123.2* ± 11.0	100.8 ± 3.8	75.8*# ± 3.7	99.2 ± 12.8	101.1 ± 5.4
Mitochondrial UCP3	167.4* ± 15.8	99.5 ± 2.9	135.5* ± 2.8	175.5*# ± 5.1	139.8 ± 15.7	119.1 ± 24.2
Body weight (AUC)	90.6* ± 1.2	100.7 ± 2.9	110.6* ± 1.6	100.0*# ± 1.8	115.2* ± 5.6	111.8* ± 3.1
Fat index						
Subcutaneous fat	104.4 ± 28.3	106.4 ± 24.8	386.9* ± 66.9	304.4* ± 35.8	310.4* ± 25.8	313.4* ± 35.8
Visceral fat index	99.8 ± 19.5	116.4 ± 20.1	365.8* ± 63.9	267.8*# ± 52.6	370.8* ± 27.2	361.5* ± 39.1

Data are expressed as percent control with respect to the untreated ND control. Values given are means ± SEM, with n = 10.
*Significantly different from the untreated ND control; #significantly different from the untreated HFD control ($p < 0.05$)
